# Neo-digit functional reconstruction of mutilating hand injury using transplantation of multiple composite tissue flaps

**DOI:** 10.1097/MD.0000000000004179

**Published:** 2016-07-08

**Authors:** Xiucun Li, Jianli Cui, Suraj Maharjan, Xin Yu, Laijin Lu, Xu Gong

**Affiliations:** Department of Hand and Foot Surgery, The First Hospital of Jilin University, Changchun, Jilin, P.R. China.

**Keywords:** composite tissue flap transplantation, functional reconstruction, mutilating hand injury

## Abstract

**Purpose:**

Functional reconstruction of mutilating hand injuries poses a challenge to the surgeon. We present our experience with use of multiple composite tissue flaps transplant for functional reconstruction of hand in patients with mutilating hand injuries. The associated merits and demerits of these surgical approaches are briefly discussed.

**Methods:**

From August 2004 to October 2014, functional reconstruction of hand with transplantation of multiple composite tissue flaps was performed in 8 patients. These included the toe with dorsal pedis artery flap, the reverse posterior interosseous artery flap, and the anterolateral thigh flap. Mean interval from injury to functional reconstruction was 10.6 days.

**Results:**

All transplanted skin flaps and reconstructed neofingers survived completely. Only 1 patient developed wound infection at the recipient site (hand), which resolved without any debridement or revision surgery. At the donor site (foot), partial skin necrosis was observed in 1 patient, which healed with local wound care. In other patients, all wounds healed without any complications. The average range of movement at the neofinger metacarpophalangeal and interphalangeal joints was 38° and 73°, respectively. None of the patients required revision surgery.

**Conclusion:**

Use of negative pressure wound therapy and multiple composite tissue flap transplantation appears to be an effective strategy for hand functional reconstruction in patients with mutilating hand injuries. Among the multiple composite tissue flaps, use of toe transplantation combined with reverse posterior interosseous artery flap appears to be the best option.

## Abstract 2

Supplemental Digital Content is available in the text

## 1 Introduction

Mutilating hand injuries are commonly encountered and are usually the result of various complex traumas such as mangling, crush, or avulsion injury. Owing to the traumatic nature and the exquisitely interconnected anatomy of the hand, these injuries can lead to the loss of hand function and lower the quality of daily life.^[1]^ Reconstruction and restoration of function following mutilating hand injury poses a severe challenge to the reconstructive surgeon.^[2,3]^

In the past, most patients with mutilating hand injuries could be treated using a well-judged finger or palm terminalization procedure or stump revision.^[4]^ With the advances in microsurgical techniques, reimplantation of amputated fingers or palm is technically feasible, and various flaps and toe-to-hand transplant surgery techniques have been described for the functional reconstruction of mutilating hand injuries to date.^[1,3,5–7]^ In these methods, toe-to-hand transplantation combined with perforator flap is often the best solution for optimal functional restoration. However, the conventional practice is to cover the defect of mutilating soft tissue with various flaps (pedicled or free), and then the reconstruction of hand function (toe-to-hand transplantation) is performed after a period following primary surgery (definitive coverage of the defect).^[1,8–10]^ The disadvantages of this approach (staging treatment) include a prolonged treatment cycle, increased economic burden on the patients, pain associated with multiple surgical procedures, and lower quality of daily living. In addition, primary (emergency) hand functional reconstruction has many shortcomings including extensive soft-tissue defects, propensity to postoperative infection, and transplanted tissue flaps necrosis, which may be associated with the contaminated wound and traumatic tissue that has not been debrided thoroughly and completely.^[10,11]^

In order to overcome the aforementioned shortcomings, our treatment strategy for mutilating hand injuries is to perform primary reimplantation of amputated fingers provided that certain criteria are met. In all other cases, negative pressure wound therapy is applied to the amputation stump. Subsequently, multiple composite tissue flaps are used for functional reconstruction of hand with use of free toe transplantation with dorsal pedis artery flap. Furthermore, the reverse posterior interosseous artery (PIA) flap or other free tissue flaps are used to repair the soft-tissue defect of the stump.

The purpose of this study is to present our experience with functional reconstruction surgery for mutilating hand injuries with use of multiple composite tissue flap transplants. The merits and demerits of this method are summarized.

## 2 Patients and methods

This retrospective study was approved by the institutional review committee and ethics committee at the First Hospital of Jilin University. Written informed consent was obtained from all patients.

**Figure 1:**
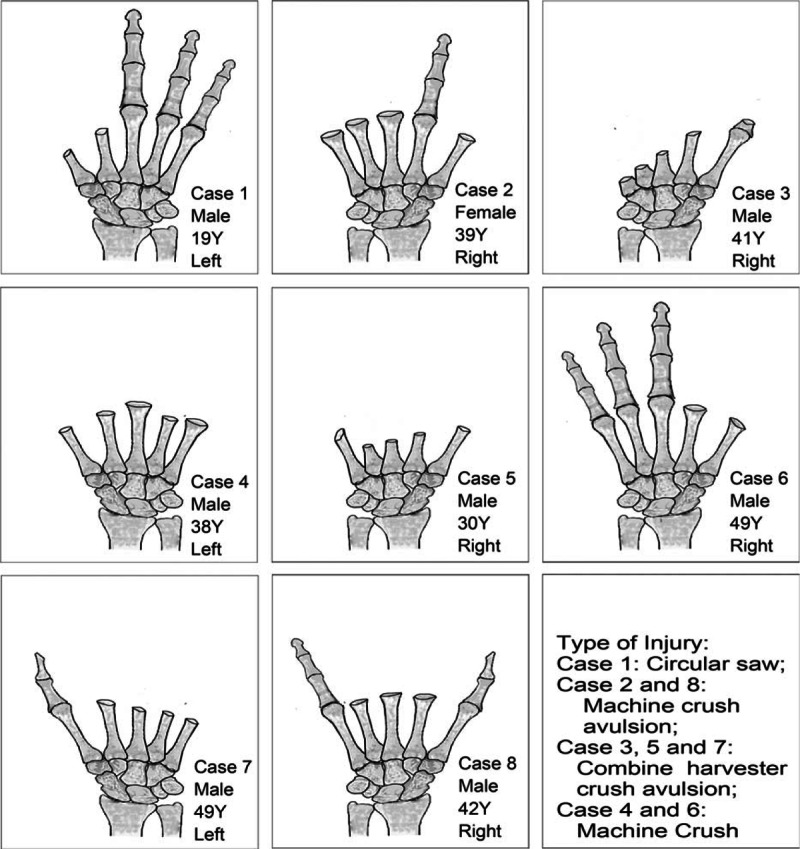
Patient characteristics and extent of injury in 8 patients.

Inclusion criteria were as follows: mutilating hand injuries resulting in amputation of 2 or more fingers and/or palm, extensive soft-tissue devitalization and defects, compromised vascular supply, significant functional impairment, and 2 or more composite tissue flaps transplantation were performed simultaneously. Accordingly, we reviewed hospital records, and found that functional reconstruction using multiple composite tissue flap transplantation had been performed in 8 patients (7 men and 1 woman) from August 2004 to October 2014. The mean age was 38 years (range, 19–49). There were 3 left hands and 5 right hands with injuries. Toe transplantation combined with reverse PIA flap for neofinger reconstruction was carried out in 4 patients. In all other cases, toe transplantation combined with grafts from other tissues was performed (Table 1). All cases were those of unilateral mutilating hand injuries and involved amputation of at least 2 fingers. Figure 1 shows patient characteristics and extent of injury in these 8 patients. Seven out of the 8 patients were followed up, mean follow-up duration was 22.6 months (range, 13–36). One patient was lost to follow up.

**Table 1:**
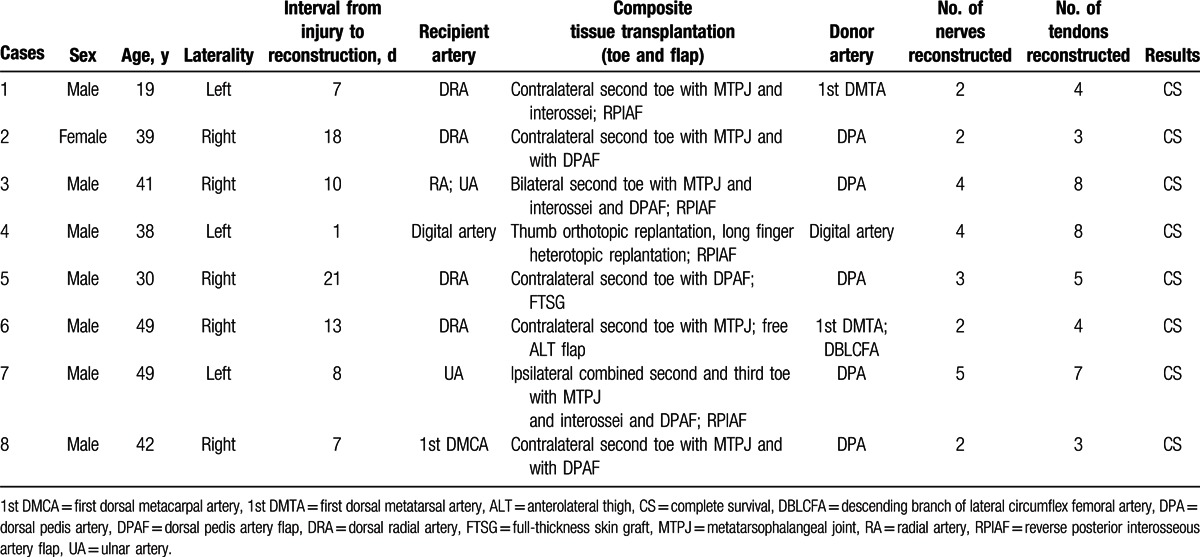
Case details of patients who underwent functional reconstruction surgery for mutilating hand injuries.

### 2.1 Reconstruction technique

The principles of reconstruction in mutilating hand injuries are thorough and complete debridement, vascular restoration, stable bony fixation, repair of specialized tissue such as tendon and nerve, followed by definitive soft-tissue coverage and reconstructive function.^[1,3]^

**Figure 2:**
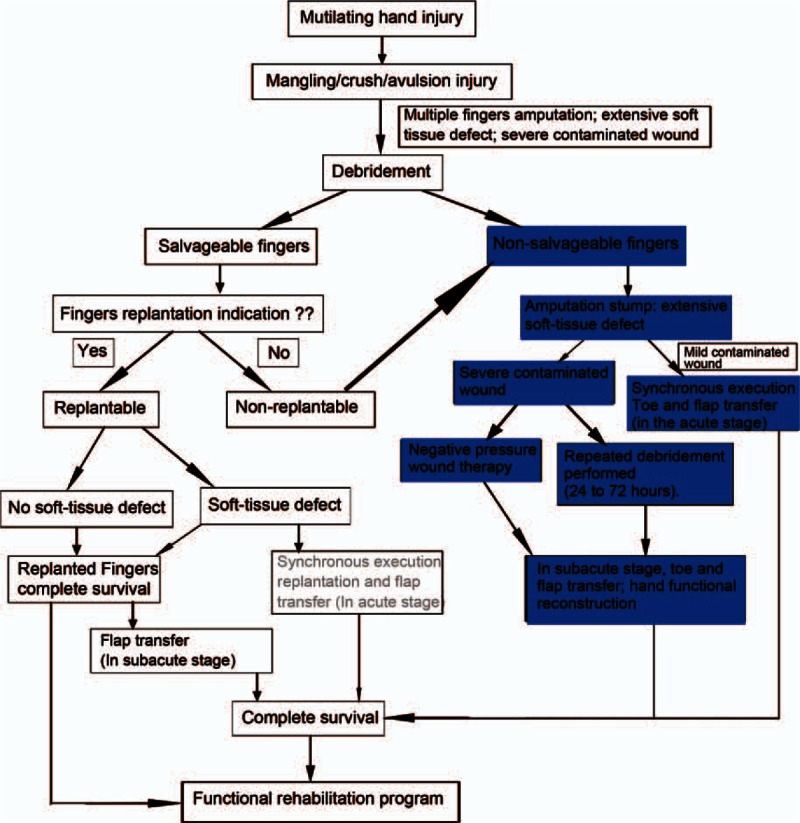
Flow diagram illustrating the steps and decision rules for functional reconstruction of hand after mutilating hand injury.

**Figure 3:**
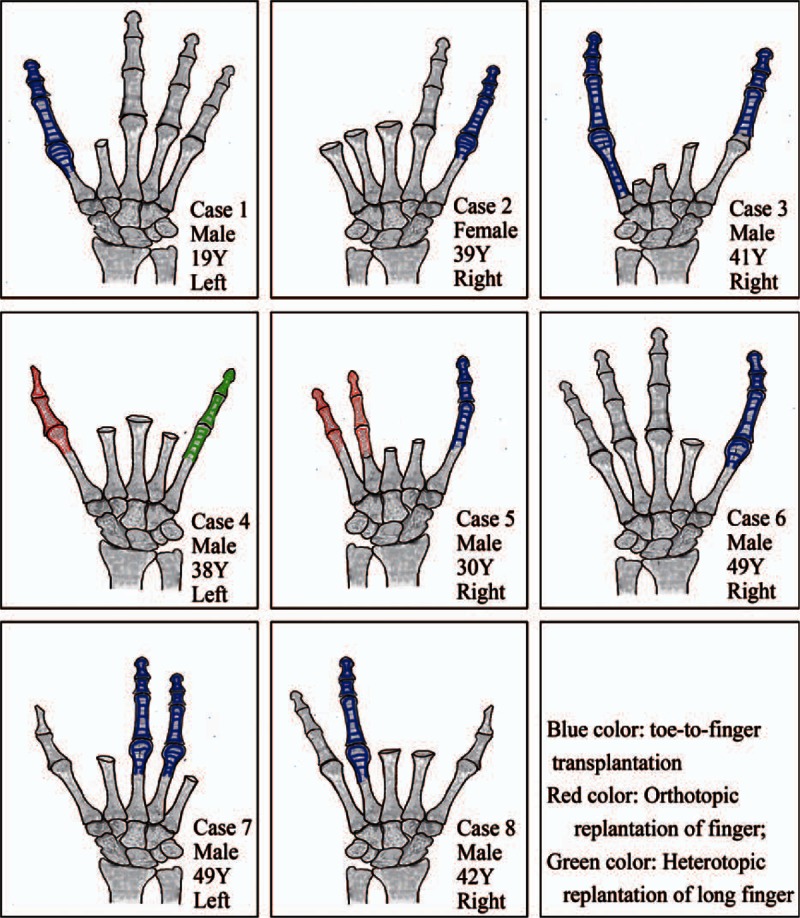
Schematic illustration of the outcomes of functional reconstruction of mutilating hand injuries.

Finger reimplantation was attempted in patients 4 and 5. The wound soft-tissue defect in patient 4 was repaired using the reverse PIA flap. In other patients, debridement and negative-pressure wound therapy was administered as primary treatment. For patients with severely contaminated wound, secondary debridement was also performed at 24 to 72 h postoperatively. Figure 2 shows the steps and decision rules for functional reconstruction after mutilating hand injury. All patients had extensive soft-tissue defects with severe loss of hand function. According to “the new reconstructive ladder,”^[12]^ finger reconstruction and repair of soft-tissue defects should be performed simultaneously and synchronously. Table 1 summarizes the details of functional reconstruction for each patient. Outcomes of finger reconstruction are shown in Fig. 3.

The methods used for harvesting of multiple composite tissue are described below: The toe along with dorsalis pedis artery flap was harvested. The venous system of the toe with dorsal pedis artery flap includes 2 major veins. Flaps were used to cover the parts with major tissue (bone, tendon, and neurovascular bundle) exposure. The flexor tendon sheath at the metatarsophalangeal joint level should not be left exposed, otherwise adhesions may result. When the metatarsophalangeal joint and interossei were included, the length of the metatarsal bone required for the graft usually ranged between 2 and 5 cm. Nerves of the toe were dissected up to the length necessary to join with the hand sensory nerves. The extrinsic tendons were dissected up to suitable lengths. According to the soft-tissue defect size and vascularity at the recipient site, reverse PIA flap or free tissue flap was transplanted. The harvesting of the flap should comply with the 3 principles of “pivot or central point, axial line, and plane.”

After preparation of the recipient site in all patients, osteosynthesis was performed with use of a single or double K-wire, followed by neurovascular end-to-end anastomoses with 9–0 nylon suture under operating microscope. The recipient sites and donor sites were closed with 4–0 nonabsorbable suture. Full-thickness skin grafts were used for closure of donor site wherever necessary.

### 2.2 Postoperative management

Postoperative care and monitoring were done for first 5 postoperative days. Anticoagulation with heparin sodium was initiated intraoperatively and continued until the 4th postoperative day. Patients were ambulated on the 10th postoperative day but were advised against any strenuous exercise for a period of 3 weeks. All skin sutures were removed 2 weeks after operation, following which cautious active hand movements were encouraged, except in cases where internal fixation was performed. K-wires were removed 4 to 6 weeks after surgery followed by functional rehabilitation program.

### 2.3 Outcome evaluation and statistical analysis

On the 10th postoperative day, the surviving neofingers and flaps were warm, pink in color, and showed a good capillary refill. At the final follow-up, sensibility of the neofinger and flap was assessed by static 2-point discrimination test.^[13]^ Grip strength was assessed using an electronic hand dynamometer (CAMRY, Guangdong Senssun Weighing Apparatus Group Ltd, Guangzhou China). Range of motion (ROM) of neofinger was measured with a goniometer. For measurement of ROM, the proximal interphalangeal joint and the distal interphalangeal joint were deemed as 1 interphalangeal (IP) owing to the small size of the intermediate and distal phalanges of the neofinger. The web span between thumb and its adjacent digit was measured with the volar surface of the hand placed flat on the table and with the thumb and its adjacent digits in maximal abduction.^[14]^ All measurements were compared with those of the contralateral normal hand.

Pain sensation at the recipient and donor sites was measured on the visual analog scale, which consisted of a 10 cm line that was divided into 3 categories: mild (0–3 cm), moderate (4–6 cm), and severe (7–10 cm).^[15]^ Cold intolerance of the neofinger and flap was evaluated using the Cold Intolerance Severity Score questionnaire.^[16]^ The maximum score was 100 and was subdivided into 4 categories: mild (0–25), moderate (26–50), severe (51–75), and extreme severity (76–100). Evaluation of hand function was performed using the Sollerman hand function test,^[17]^ which consisted of 20 subtests. Each subtest pertained to a task and which was scored on a scale of 0 to 4: 0 (not performing at all), 1 (incomplete performance [in <60 s]), 2 (with great difficulty [40–60 s]), with slight difficulty [20–40 s]), without any difficulty [in 20 s]). The aggregate score ranged from 0 to 80 points. A normal dominant hand should score 80 points and the contralateral hand 77 to 80 points. Patient satisfaction with functional recovery of the injured hand was assessed using Michigan Hand Outcomes Questionnaire, and results measured on a 5-point response scale.^[18]^

The final outcomes of each patient were documented as continuous variable, which were described by mean and range.

## 3 Results

In our case series, the mean interval from injury to functional reconstruction was 10.6 days (range, 1–21). One patient developed wound infection at the recipient site (hand), which resolved without the need for surgical debridement. Partial skin necrosis at the donor site was observed in 1 patient, which healed with local wound care. In other patients, all wounds healed without any complication. All the flaps and the neofingers remained viable. Further, none of the patients developed any signs of venous congestion in the affected parts.

Outcomes evaluated at the final follow-up are summarized in Table 2. The mean static 2-point discrimination of the neofinger and flap was 8.2 mm (range, 6.6–10.2) and 16.2 mm (range, 14.7–18), respectively. Mean active movement at the metacarpophalangeal and IP joints of the neofingers was 38° (range, 20° to 50°) and 73° (range, 37° to 88°), respectively. The corresponding mean range of movement on the contralateral side was 67° (range, 55° to 86°) and 99° (range, 95° to 104°), respectively. The average grip strength of the injured and contralateral hand was 18.9 kg (range, 1.8–31.6) and 34.2 kg (range, 25.3–40.6), respectively. The average Sollerman hand function test scores for the injured and the contralateral hand were 66 (range, 32–75) and 79 (range, 77–80), respectively. The mean span of the first web was 9.4 cm (range, 6.1–13.5 cm). The visual analog scale score for pain in patients 7 and 3 were 1 and 2, respectively. In terms of Cold Intolerance Severity Score, patient 3 showed mild cold intolerance. No instances of scale contracture were reported during the follow-up period. The donor sites had no functional impairment. Further, none of the patients required any revision surgery. As assessed with the Michigan Hand Outcomes Questionnaire for the appearance of the reconstructed hand, 5 patients were very satisfied with the outcomes (score 5), 1 patient was satisfied (score 4), and 1 patient indicated a general satisfaction (score 3).

**Table 2:**
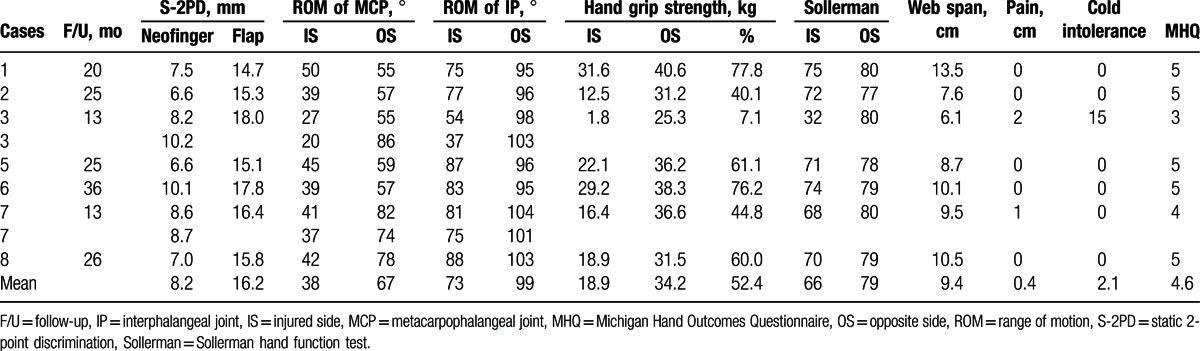
Outcomes of functional reconstruction of mutilating hand injuries on follow-up.

### 3.1 Case 1 (patient no. 3)

**Figure 4:**
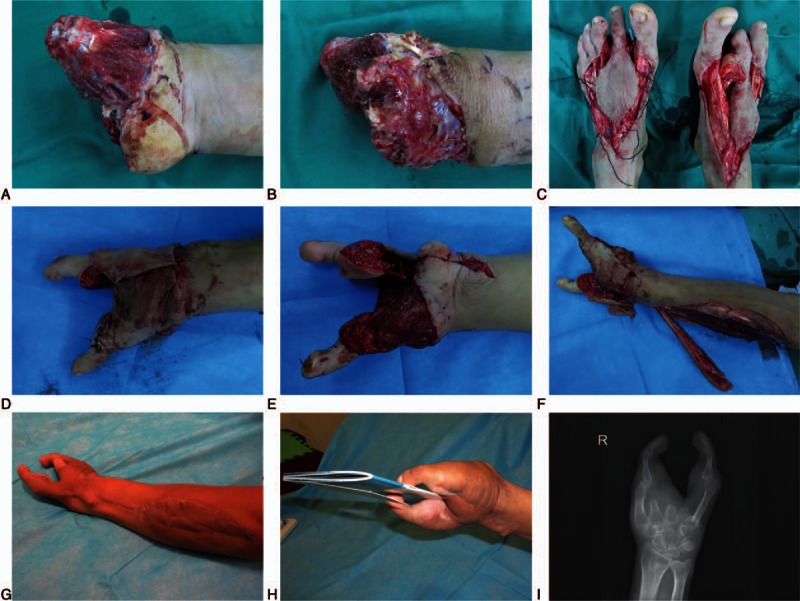
Preoperative, intraoperative, and follow-up pictures of patient 3. Picture of hand showing (A and B) the extent of injury; (C) the dissection of bilateral second toe with dorsal pedis artery flap; (D) the dorsal aspect of hand; (E) the volar aspect of the injured hand; (F) the reverse posterior interosseous artery flap; and (G–I) the results after 13 mo.

A 41-year-old male farmer suffered from a crushing avulsion injury of the right hand while operating a combined harvester. All fingers were amputated. After wound debridement, the stump was found to have extensive soft-tissue defects (Fig. 4A and B). Bilateral second toe with dorsal pedis artery flaps were used for functional reconstruction of the hand while the soft-tissue defect of amputation stump was covered by the reverse PIA flap (Fig. 4C–F). As assessed in the 13th postoperative month, patient was very satisfied with the function and appearance of the reconstructed finger (Fig. 4G–I, and Video 1).

### 3.2 Case 2 (patient no. 5)

**Figure 5:**
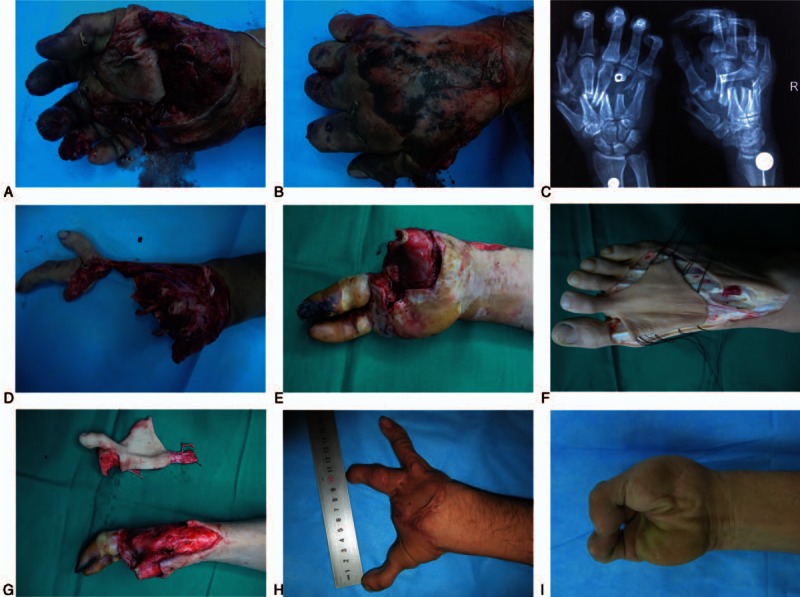
Preoperative, intraoperative, and follow-up pictures of patient 5. Picture of the injured hand showing the extent of (A and B) the injury; (C) the X-ray radiograph of the right hand; (D) the wound after debridement; (E) the surviving ring and little fingers; (F and G) dissection of the second toe with dorsal pedis artery flap; and (H and I) the results after 25 mo.

A 30-year-man sustained a crushing avulsion injury of the right hand with amputations of all fingers at the intermediate level of metacarpus (Fig. 5A–C). The injury was sustained from combined harvester at work. There were extensive soft-tissue defects in the hand (Fig. 5D). After debridement, orthotopic reimplantation of the ring and little finger was performed. Replantable fingers retained their viability at 1 week after surgery (Fig. 5E). The patient underwent hand functional reconstruction using the contralateral second toe with a dorsal pedis artery flap (Fig. 5F). The soft-tissue defect of stump was covered by full thickness skin graft. The neofinger and flap survived completely. The patient was very satisfied with the functional and esthetic restoration of finger as assessed at 25 months after surgery (Fig. 5H and I; Video 2).

## 4 Discussion

Currently, there is no consensus on the approach to management of mutilating hand injuries. Several options exist for treatment of mutilating hand injuries. These include finger or palm amputation, reimplantation, and microsurgical functional reconstruction. In severe cases, careful assessment of the wound and the amputated segments will help in planning the treatment. Nonreplantable parts should also be thoroughly inspected and the feasibility of their use for hand functional reconstruction assessed.^[19]^ The treatment approach to mutilating injuries of hand involves detailed customized planning based on the individual characteristics. The outcomes of hand functional reconstruction also depend on the mechanism of injury.^[20]^

Mutilating hand injuries often include amputation of multiple fingers and/or palm with extensive soft-tissue defects, compromised vascular supply and significant functional impairment. The viability of different injured tissues often varies. Wound contamination with foreign bodies and microorganisms is a common accompaniment of such injuries. Despite adequate debridement, wound infection is a common complication. Secondary debridement is often required at 24 to 72 h after surgery.^[6,21]^ According to “the new reconstructive ladder,”^[12]^ negative pressure wound therapy of the amputation stump increases the rate of granulation tissue formation; decreases peri-wound edema, infection; and shortens the time to closure.^[22,23]^

Timing of functional reconstruction of mutilating hand injuries is a controversial issue.^[24]^ According to Harrison et al, the timing of reconstruction has no significant effect on postoperative outcomes,^[25]^ while Derderian et al^[26]^ proposed the time window between 6 and 21 days after injury as being optimal for microvascular-free tissue transplantation for hand functional reconstruction. Others are in favor of delaying the reconstruction until the subacute stage,^[27,28]^ while Brenner et al^[21]^ have favored functional reconstruction in the acute stage (time window from 24 h after injury to 3 days after injury). The time window between 3 days and 3 weeks after injury is commonly referred to as the subacute stage. The evolution of perioperative management and use of negative pressure wound therapy allow for secure reconstruction along with debridement in the subacute stage. We believe that the optimal time for functional reconstruction should be guided by the extent of tissue injury, edema, presence of wound infection, and whether critical neurovascular bundle is exposed. The infection and flap-related adverse outcomes tend to be lower with reconstruction in the subacute stage by which time multiple debridement may be performed. Subsidence of inflammation and growth of fresh granulation tissue is often the optimal time for reconstruction. Fresh granulation tissue usually grows in 2 to 3 days after tissue injury, and therefore, we are in favor of performing reconstruction in the subacute stage.

With regard to the treatment of mutilating hand injuries, the reverse on-top plasty,^[29]^ the free fillet flap after traumatic amputation,^[30]^ and toe-to-antebrachial stump transplantation^[31]^ were carried out and had satisfactory outcomes. But these methods are difficult to compare because there is no uniform standard of treatment on mutilating hand injuries. However, the goal of treatment is to maximize the recovery of hand function. In our series, we achieved good outcomes with use of multiple composite tissue flaps for reconstruction of the amputation stump in the subacute stage. It may reduce the risk of wound infection and increase the success rate of surgery. Such an approach reduces the need for multiple operations, shortens the length of hospital stay, lowers the costs of treatment, and allows for early return of the patient to his routine daily activities. Early rehabilitation training is necessary to prevent tendon adhesion, to reduce post-traumatic edema, and to maximize the functional recovery of hand. In our series, it was of vital importance to reserve sufficient length of the skeleton, tendon, and neurovascular bundles during the debridement procedure, in order to allow flexibility in the subacute phase of the reconstruction, all of which were performed with careful and meticulous debridement. In all patients except in the case of patient 4 (Table 1, Fig. 3), toe-to-hand transplantation with dorsal pedis artery flap was considered a priority. To this end, the soft-tissue defects were covered using the reverse PIA flap or other tissue grafts.

The advantages of functional reconstruction using toe transplantation combined with use of the reverse PIA flap are given below. In comparison to the anterolateral thigh flap, the reverse PIA flap is thin, pliable, and hairless, and has a texture akin to that of the skin of hand. Moreover, the reconstructed hand does not usually require a revision surgery, which reduces the overall time duration for functional reconstruction, and the associated cost of treatment. Moreover, use of full thickness skin graft at the amputation stump is associated with a lower survival rate and is liable to result in tendon adhesion. Tendon adhesions tend to reduce the range of flexion and extension movements of the neofinger. On the flip side, the skin graft used at the donor site of the PIA flap may affect the postoperative venous drainage. Nonetheless, the skin graft affected the esthetic value of the forearm since it is usually exposed. There is a need for more surgeons trained in these techniques, and larger studies to develop these procedures as a viable option in hand surgery.

The main limitation of this study is the small number of patients, which did not allow robust statistical analyses, and also may have resulted in a sampling error. The different durations of follow-up for evaluating the final outcome might have led to statistical errors. The results of this study require validation in a larger set of patients, preferably in a multicenter study.

## 5 Conclusion

Functional reconstruction of mutilating hand injury is a challenging task. Although there is a general lack of consensus on the optimal time window for reconstructive surgery, we believe that functional reconstruction with use of multiple composite tissue flaps is best performed in the subacute stage. The advantages include a lower risk of wound infection, increased chances of favorable surgical outcomes, shortened length of hospital stay, cost savings, and an early return of the patient to daily activities. For the multiple composite tissue flaps, toe transplantation combined with the reverse PIA flap is the best strategy in our experience.

## Supplementary Material

Supplemental Digital Content

## Supplementary Material

Supplemental Digital Content
